# Supplementary material for: The causal relationship between the human gut microbiota and pyogenic arthritis: a Mendelian randomization study
Source: Front Cell Infect Microbiol. 2024 Nov 26;14:1452480. doi: 10.3389/fcimb.2024.1452480 (PMC11629706; doi:10.3389/fcimb.2024.1452480)
Supplement: Supplementary file 1 [file DataSheet1.zip › mendelian test/ebi-a-GCST90027716.csv_leave_one_out.pdf]

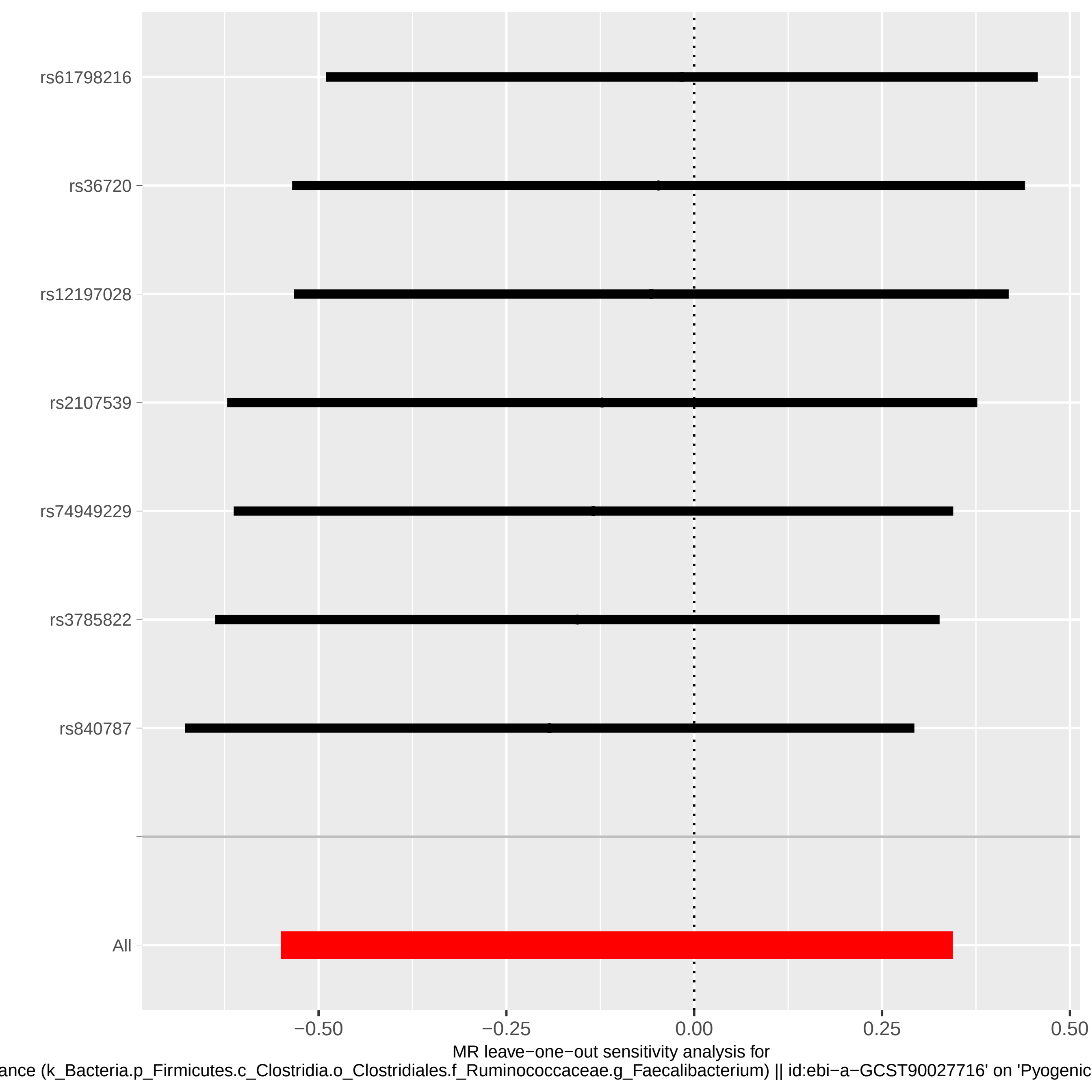
rs61798216
rs36720
rs12197028
rs2107539
rs74949229
rs3785822
rs840787
All
−0.50
−0.25
0.00
0.25
0.50
MR leave−one−out sensitivity analysis for
ance (k_Bacteria.p_Firmicutes.c_Clostridia.o_Clostridiales.f_Ruminococcaceae.g_Faecalibacterium) || id:ebi−a−GCST90027716' on 'Pyogenic
